# Supplementary material for: Evaluation of morphological variations of mandibular bone in adult bruxers using CBCT: A cross-sectional study
Source: PLoS One. 2026 Feb 5;21(2):e0342472. doi: 10.1371/journal.pone.0342472 (PMC12875488; doi:10.1371/journal.pone.0342472)
Supplement: S4 Table — (PDF) [file pone.0342472.s005.pdf]

S4 Table. Descriptive statistics for the non-bruxer group (68 mandibles).

ROI	Minimum (HU)	Q1 (HU)	Median (HU)	Mean (HU)	Standard deviation (HU)	Q3 (HU)	Maximum (HU)	Missing data (percentage and number)
31	110.7	429.3	614	618.3	223.85	783	1029.5	0
33	164.2	358.3	440.5	512.3	225.37	667.1	1325.6	0
34	58.61	256.28	379.66	411.59	196.43	568.79	839.02	2 (2.9%)
36	-84.65	111.06	186.66	261.76	248.5	383.63	1039.16	5 (7.4%)
41	182.6	475.1	652.5	622.5	199.4	757.3	1164.4	1 (1.4%)
43	25.49	387.34	515.91	530.76	220.52	636.31	1162.64	1 (1.4%)
44	-8.18	278.91	394.41	423.37	190.72	500.53	1031.35	0
46	10.42	140.62	218.44	281.55	235.26	391.45	1322.1	3 (4.4%)

ROI: Region Of Interest. Q1: first quartile. Q3: third quartile. HU: Hounsfield units.
