# Supplementary material for: Evaluation of morphological variations of mandibular bone in adult bruxers using CBCT: A cross-sectional study
Source: PLoS One. 2026 Feb 5;21(2):e0342472. doi: 10.1371/journal.pone.0342472 (PMC12875488; doi:10.1371/journal.pone.0342472)
Supplement: Attachment — Submitted filename: Response to Reviewers.docx [file pone.0342472.s010.docx]

Response to Reviewers

**Academic Editor**

Dear Editor,

Thank you for your review of our manuscript.

We have modified the items you mentioned in your feedback. You will find our responses to each of your suggestions in blue.

Kind regards,

The authors

1. Please ensure that your manuscript meets PLOS ONE's style requirements, including those for file naming.

The requested checks have been carried out, and changes made to figure legends and supporting information.

2. Please include your full ethics statement in the ‘Methods’ section of your manuscript file. In your statement, please include the full name of the IRB or ethics committee who approved or waived your study, as well as whether or not you obtained informed written or verbal consent. If consent was waived for your study, please include this information in your statement as well.

In accordance with French regulations, single-center retrospective studies using pre-existing data for research purposes do not require written consent from patients, provided they have been informed in advance and do not object, and subject to a favorable opinion from an ethics committee.

This indication has been added to the manuscript (lines 99-100).

3. We note that Figures 1 and 2 in your submission contain copyrighted images. All PLOS content is published under the Creative Commons Attribution License (CC BY 4.0), which means that the manuscript, images, and Supporting Information files will be freely available online, and any third party is permitted to access, download, copy, distribute, and use these materials in any way, even commercially, with proper attribution.

- Figure 1 is based on a screenshot from the 3D Slicer software, which is a free open source software distributed under a BSD style license. The license does not impose restrictions on the use of the software, as indicated on the software website at the following address: https://www.slicer.org/commercial-use.html

This figure has therefore been retained in the manuscript.

- Figure 2 has been removed from the manuscript.

4. We note that there is identifying data in the Supporting Information files S2_Table.csv, S3_Table.csv, S4_Table.csv, S5_Table.csv, and S6_Table.csv. Due to the inclusion of these potentially identifying data, we have removed this file from your file inventory. Prior to sharing human research participant data, authors should consult with an ethics committee to ensure data are shared in accordance with participant consent and all applicable local laws.

Thank you for these clarifications. The necessary modifications have been made.

**Reviewers**

Dear Reviewers,

Thank you for your careful review of the manuscript and your suggestions for improving its quality.

We have considered every one of your comments to improve this article. You will find our responses to your feedback in blue.

Kind regards,

The authors

**Reviewer #1**

1. Introduction and Literature Review

The introduction could benefit from a clearer identification of the gap in the literature. While panoramic radiographs have been used previously, the novelty and clinical relevance of employing CBCT in this specific context should be more explicitly stated.

Thank you for your comment. It has been added (lines 79–82). In addition, this is developed in the discussion (lines 256-260).

The definition of bruxism is accurate, but a brief clarification on the difference between awake and sleep bruxism—and whether this distinction was considered—would enhance the reader’s understanding.

We have taken your suggestion into account to complete the manuscript (lines 59-60). As this distinction could not be taken into account due to the absence of this data in a certain number of medical records, it could not be exploited in this study. This element has been added to the discussion (line 313).

2. Methodological Clarifications

Diagnostic Criteria for Bruxism: One of the main limitations of the study is the absence of a standardized diagnostic protocol for identifying bruxers. Please clarify whether diagnosis was based solely on clinical records, self-report, clinical signs (e.g., tooth wear), or validated instruments. If diagnosis was retrospective and inconsistent, this should be clearly emphasized as a limitation.

Thank you for your comment. This was clarified in the inclusion criteria (lines 106-108).

Sample Size Calculation: Although a sample size calculation is presented, it is unclear whether it was based on the primary outcome measures (bone density or mandibular angle). Please specify the statistical parameters and assumptions used.

Thank you for your comment. The requested information has been added (lines 125-129).

3. Discussion and Interpretation

Clinical implications of the findings—particularly regarding implant planning, orthodontics, or risk of mandibular changes—should be more explicitly discussed.

Your comments have been taken into account, and the clinical implications are detailed lines 283-292 and 326-329.

The association between decreased mandibular angle and bruxism is noteworthy. Please elaborate on whether this may reflect muscular hyperactivity, skeletal remodeling, or growth-related factors.

These elements have been mentioned lines 322-325.

The lack of control for potential confounding variables (e.g., sex, occlusal classification, medication use, parafunctional habits) should be acknowledged more directly.

Thank you for your comment. Details of confounding variables have been added line 336.

**Reviewer #2**

1. Sample size calculation:

The sample size was calculated based on prevalence estimation (22%) and a 7% margin of error, which is not appropriate for a comparative study involving continuous variables. I recommend recalculating the sample size based on:

-A clinically relevant minimum difference between groups;

-An estimated standard deviation (from pilot data or literature);

-A significance level of α = 0.05;

-Statistical power of at least 80% (1-β ≥ 0.8).

Thank you for this comment and the associated information. The calculation of the number of subjects required has been corrected according to your indications. It is based on an expected difference between groups of 200HU, with a standard deviation of 250HU, an alpha risk of 0.05% and a power of 90% (lines 125-129).

However, in order to anticipate the potential loss of data (missing teeth, missing mandibular angle), a number greater than the minimum size required was included, to guarantee sufficient statistical power for both parts of the study, particularly for the mandibular angle section.

2. Bruxism diagnosis:

I suggest clarifying the criteria used to classify subjects as bruxers or non-bruxers, as recorded in the medical charts. The absence of standardized or validated diagnostic tools (such as STAB) introduces classification bias, which should be clearly acknowledged as a limitation. If possible, I encourage providing descriptive data on bruxism subtypes (sleep vs. awake, centric vs. eccentric), which could help strengthen the clinical interpretation.

Thank you for your comment. Additional descriptive elements concerning the diagnosis of bruxism have been added lines 106-108.

Unfortunately, data on bruxism subtypes were not sufficiently mentioned in the medical records to allow clinical interpretations. This was discussed at the end of the manuscript.

3. Confounding factors:

There are apparent age differences between groups, yet no statistical adjustment was performed. I recommend using multivariate models (e.g., linear regression) or stratified analysis to control for potential confounders such as age and sex. If not feasible, this limitation should be explicitly acknowledged in the discussion.

Thank you for your comment. The corresponding change has been added (line 337).

4. Excessive number of tables and data simplification:

The manuscript includes a large number of tables, many of which present redundant or overly granular data. I suggest consolidating tables where possible, grouping by anatomical region (e.g., symphyseal, premolar, molar areas), and simplifying the presentation to improve clarity.

Thank you for your comment.

The presentation of the results has been modified (lines 198-209). To improve readability and avoid repetition, 2 tables have been removed from the manuscript, and are now part of the supporting information.

We preferred to keep the values in a single table to give the reader an overview of the results. We hope the result is more in line with your expectations.

5. Clinical relevance of findings:

Beyond statistical significance, I suggest discussing whether the observed differences (in HU and degrees) are clinically meaningful and how they might influence treatment planning in implantology or orthodontics.

Thank you for your comment. Modifications has been added lines 282-292 and 326-329.

7. Age stratification (≤45 vs. >45 years):

If the division by age group was applied in the tables or analysis, I suggest justifying the rationale for this cutoff. If no statistical comparison was performed based on age, this stratification should be removed or explained as purely descriptive.

Thank you for your comment. The division by age group was purely descriptive. The cut-off of 45 years old was the median age of the patients included in the study. This clarification has been added to the manuscript (line 189).
