# Supplementary material for: Evaluation of morphological variations of mandibular bone in adult bruxers using CBCT: A cross-sectional study
Source: PLoS One. 2026 Feb 5;21(2):e0342472. doi: 10.1371/journal.pone.0342472 (PMC12875488; doi:10.1371/journal.pone.0342472)
Supplement: Attachment — Submitted filename: Response_to_Reviewers_auresp_2.docx [file pone.0342472.s011.docx]

Response to Reviewers

**Reviewer #1**

Reviewer #1: All comments are completely answered. Thank you for opportunity to review. This is a very good study. The authors are to be congratulated for their research and should feel encouraged to increase the sample size in future investigations.

We would like to thank you for your feedback and the valuable advice you gave us to improve the quality of the manuscript.

**Reviewer #2**

Overall, the manuscript has been improved, and most of the limitations highlighted in the previous review have been adequately addressed or properly justified. The study presents relevant findings, but I suggest a few additional revisions before acceptance:

-Review of references

The citation Lumetti S. et al., 2016 [9] does not adequately support the statement in lines 57–58: “can weaken the teeth and the temporomandibular joints, and generate mastication myalgia [9]”. Please revise this reference and consider replacing it with a more appropriate source.

Thank you for your comment. This citation has been replaced by a more appropriate reference.

Matusz K, Maciejewska-Szaniec Z, Gredes T, Pobudek-Radzikowska M, Glapiński M, Górna N, Przystańska A. Common therapeutic approaches in sleep and awake bruxism - an overview. Neurol Neurochir Pol. 2022;56(6):455-463. doi: 10.5603/PJNNS.a2022.0073. Epub 2022 Nov 29.

-Study limitations

Although the limitations are discussed, I recommend moving the content currently found in lines 316–329 into the Discussion as the final paragraph. Alternatively, a specific subsection entitled “Study limitations and recommendations for future research” could be created.

Thanks to your suggestion, a section “Study limitations and recommendations for future research” has been created at the end of the discussion (line 315).

-Conclusion

Please ensure that the Conclusion is concise and directly answers the research question: whether differences exist between bruxers and non-bruxers, and in which variables these differences were found.

Thank you for your comment. The conclusion has been rewritten according to your instructions (lines 333-339).
