# Supplementary material for: Evaluation of morphological variations of mandibular bone in adult bruxers using CBCT: A cross-sectional study
Source: PLoS One. 2026 Feb 5;21(2):e0342472. doi: 10.1371/journal.pone.0342472 (PMC12875488; doi:10.1371/journal.pone.0342472)
Supplement: Attachment — Submitted filename: Response_to_Reviewers_auresp_3.docx [file pone.0342472.s012.docx]

Response to Reviewers

**Reviewer #1**

Reviewer #1: Thank you for all corrections and good luck in your research. It’s a very complex and necessary research

We would like to thank you for your feedback to improve the quality of the manuscript.

**Reviewer #2**

Reviewer #2: I congratulate the authors once again on the development of this work. All recommendations have been accepted and addressed. During the process of reviewing this article, an updated concept of bruxism was published. I simply recommend that the new concept be incorporated into your work. The reference is as follows: Verhoeff MC, Lobbezoo F, Ahlberg J, Bender S, Bracci A, Colonna A, Dal Fabbro C, Durham J, Glaros AG, Häggman-Henrikson B, Kato T, Koutris M, Lavigne GJ, Nykänen L, Raphael KG, Svensson P, Wieckiewicz M, Manfredini D. Updating the Bruxism Definitions: Report of an International Consensus Meeting. J Oral Rehabil. 2025 Sep;52(9):1335-1342. doi: 10.1111/joor.13985. Epub 2025 May 1. PMID: 40312776; PMCID: PMC12408978

Thank you for your comment and your interest in this work. This citation has been added to update the bibliography.
